# Herbal remedy clinical trials in the media: a comparison with the coverage of conventional pharmaceuticals

**DOI:** 10.1186/1741-7015-6-35

**Published:** 2008-11-26

**Authors:** Tania Bubela, Heather Boon, Timothy Caulfield

**Affiliations:** 1 School of Public Health, Clinical Sciences Building, University of Alberta, Edmonton, Alberta, T6G 2G3, Canada; 2 Leslie Dan Faculty of Pharmacy and Health Policy, Management and Evaluation, Department of Family and Community Medicine, Faculty of Medicine, University of Toronto, Ontario, Canada; 3 Health Law Institute, Faculty of Law, School of Public Health, University of Alberta, Edmonton, Alberta T6G 2G3, Canada

## Abstract

**Background:**

This study systematically compares newspaper coverage of clinical trials for herbal remedies, a popular type of complementary and alternative medicine, with clinical trials for pharmaceuticals using a comparative content analysis. This is a timely inquiry given the recognized importance of the popular press as a source of health information, the complex and significant role of complementary and alternative medicine in individual health-care decisions, and the trend toward evidence-based research for some complementary and alternative medical therapies. We searched PubMed for clinical trials, Lexis/Nexis for newspaper articles in the UK, US, Australia/New Zealand, and Factiva for Canadian newspaper articles from 1995 to 2005. We used a coding frame to analyze and compare 48 pharmaceutical and 57 herbal remedy clinical trials as well as 201 pharmaceutical and 352 herbal remedy newspaper articles.

**Results:**

Herbal remedy clinical trials had similar Jadad scores to pharmaceutical trials but were significantly smaller and of shorter duration. The trials were mostly studies from Western countries and published in high-ranking journals. The majority of pharmaceutical (64%) and herbal remedy (53%) clinical trials had private sector funding involvement. A minority declared further author conflicts of interest. Newspaper coverage of herbal remedy clinical trials was more negative than for pharmaceutical trials; a result only partly explained by the greater proportion of herbal remedy clinical trials reporting negative results (*P *= 0.0201; *χ*^2 ^= 7.8129; degrees of freedom = 2). Errors of omission were common in newspaper coverage, with little reporting of dose, sample size, location, and duration of the trial, methods, trial funding, and conflicts of interest. There was an under-reporting of risks, especially for herbal remedies.

**Conclusion:**

Our finding of negative coverage of herbal remedy trials is contrary to the positive trends in most published research based primarily on anecdotal accounts. Our results highlight how media coverage is not providing the public with the information necessary to make informed decisions about medical treatments. Most concerning is the lack of disclosure of trial funding and conflicts of interest that could influence the outcome or reporting of trial results. This lack of reporting may impact the medical research community, which has the most to lose by way of public trust and respect.

## Background

Health care receives significant media attention, and complementary and alternative medicine (CAM) is no exception [1-3]. Given the continued public interest in CAM, this media attention is hardly surprising. Indeed, CAM is a multibillion dollar business [4]. An increasingly empowered [5] and informed public continues to turn to CAM as an alternative or supplement to conventional medical therapies [6], even though the mechanisms of action are not always well understood, efficacy is often unsupported by research, and use has been associated with adverse reactions [7-9]. There are varied explanations for the appeal of CAM, such as belief in a particular philosophical orientation towards health and life, general dissatisfaction with conventional medicine, and belief that it is natural and therefore, less harmful than biomedical treatments [10].

In this paper we explore the nature and tone of newspaper coverage of clinical trials for herbal remedies, and compare it with coverage of clinical trials for pharmaceuticals used to treat the same medical conditions. Given the recognized importance of the popular press as a source of health information [11,12], the complex and significant role of CAM in individual health-care decisions, and the trend toward evidence-based research for some CAM therapies [13-15], this seems a timely investigation. We examine, through a comparative content analysis, the quality of information provided by newspapers about one type of news story about CAM: clinical trials and their outcomes, recognizing that reporting the results of clinical trials does not represent the majority of news coverage on CAM. This type of study, comparing media coverage with the scientific research it covers is a well recognized method in media studies [16]. Is the tone of reporting different for herbal remedy versus pharmaceutical clinical trials? Are there differences in the sources of trial funding and the reporting of that issue? What about the reporting of conflicts of interest? This latter question seems particularly important as real or perceived conflicts of interest may diminish public trust in medical research [17].

## Methods

We first searched Lexis/Nexis for newspaper articles in the United Kingdom and Ireland (UK), the United States (US), and Australia/New Zealand and Factiva for newspaper articles in Canada using the search term '(herb or herbal) and remedy and "clinical trial"' from 1 January 1995 to 1 June 2005. This search strategy identified English language news articles that used both the term 'clinical trial' and 'herb/herbal'. We started with newspaper articles and not the clinical trials because few herbal remedy clinical trials receive media coverage. We retained all articles that discussed the results of an identifiable, published clinical trial. As many newspaper articles do not use the expression 'clinical trial' but instead use terms such as 'study', we also conducted specific searches on Lexis/Nexis and Factiva for additional newspaper articles on each clinical trial using different combinations of search terms, including journal name, author name(s), herbal remedy tested, and lead research institution. We omitted duplicate newspaper articles, selecting the article with highest word count, to take into account the effect of syndicated articles and newswires. We identified and collected the published clinical trials using Google searches and PubMed.

Two project-naïve, undergraduate student coders used a standardized coding frame, a common method for analyzing media coverage [18-21] for clinical trials (Appendix 1), to assess: journal name, year, type of institution where research was conducted, medical condition (using ICD-9-CM: International Classification of Diseases, 9th revision, Clinical Modification), dose, location of clinical trial, framed as controversy, benefits, risks, quality of the trial (Jadad score) [22], sample size, length of trial, conflicts of interest, trial funding, tone of clinical trial outcome, and overall tone of the discussion/conclusions, taking into account study limitations. For newspaper articles, the coders assessed the newspaper, country, year, word count, news format, themes, name of treatment, doses tested, use specified, main voice, location of clinical trial, framed as controversy, benefits, risks, conflicts of interest, funding of clinical trial, involvement of funding agency, how conflict of interest is viewed, tone of assessment of clinical trial outcomes, framing, accuracy, and, overall, whether the main claims in the newspaper article reflected the research findings.

The coders were first trained to use the coding frame by the lead author and then worked together under supervision for 2 weeks. If discrepancies arose, the coders reached a common interpretation and kept a log of decisions. The coders then individually coded the remaining articles (70% and 20%, respectively) with approximately 10% overlap to calculate inter-coder reliability, using Kappa scores. The coders reconvened periodically to discuss issues that had arisen during coding. The median of Kappa scores for clinical trials was 0.85 (range 0.39–1.00; *N *= 12) and for newspaper articles was 0.71 (range 0.47–1.00; *N *= 16), indicating good to excellent agreement. Independent variable Kappa scores were between 0.65 and 1.00.

One author (HB) with expertise in pharmacology identified corresponding pharmaceuticals for each medical condition identified in the coded herbal remedy clinical trials. We conducted searches on Lexis/Nexis and Factiva as above for newspaper coverage of identifiable clinical trials of the pharmaceuticals. As significantly more pharmaceutical trials received press coverage, we randomly selected up to three clinical trials per medical condition and then repeated the specific newspaper searches and coding methodology as above.

### Statistical analysis

We performed a classification tree analysis, using CART Pro, version 6.0 to focus our analysis on the most important variables [18]. The independent variables for clinical trials were 'overall tone' and the 'tone of clinical trial outcomes' and for newspaper articles were 'overall tone' and 'tone of assessment of the trial'. The classification tree used twoing for ranked data as the splitting method and determined which variables from the coding frame were most important in assigning a newspaper article or clinical trial to one of the three categories of tone (negative, neutral, positive). The most important variables for both measures of tone for clinical trials were length of trial, sample size, year, total number of benefits, total number of risks/costs, and Jadad score. The most important variables for both measures of tone for newspaper articles were word count, year, total number of benefits, and total number of risks/costs. We also selected additional variables of interest: for clinical trials, conflicts of interest and type of funding, and for newspaper articles, medical condition, conflicts of interest, and type of funding. National differences were not important and, therefore, the data from the different countries were combined.

We compared the distributions of the two measures of tone for clinical trials and newspaper articles between treatment type: herbal remedy or pharmaceutical using χ-squared test. We then used two sample Wilcoxon tests to compare the selected continuous variables between herbal remedies and pharmaceuticals for both clinical trials and newspaper articles. In addition, we compared the remaining variables presented in Table 1 using χ-square analysis. All tests were two-sided, and we considered *P *values of 0.05 or lower as statistically significant.

**Table 1:** Coding variables for clinical trials of herbal remedies and pharmaceuticals and associated newspaper articles

**Coding variable: clinical trials**	**Herbal remedy clinical trial** (*N *= 57)	**Pharmaceutical clinical trial** (*N *= 48)	**Statistical significance**
Mean number of benefits	1.6	1.4	NS
Mean number of risks	0.9	1.1	NS
Mean sample size	144	12,124	*P *< 0.0001
Mean duration of trial (days)	124	1435	*P *= 0.008
Mean Jadad score	3.2	3.1	NS
Dose specified	94.7%	72.9%	*P *= 0.002
Trial described as randomized	78.9%	72.9%	*P *= 0.001
Trial described as double-blind	71.9%	68.8%	*P *< 0.001
Withdrawals and dropouts described	82.5%	79.2%	NS
Conflicts of interest NOT specified	77.2%	50.0%	*P *= 0.003
Funding of trial NOT specified	33.9%	8.3%	*P *= 0.004
**Coding variable: newspaper articles**	(*N *= 352)	(*N *= 201)	
Mean word count	698	716	NS
Mean number of benefits	1.3	1.2	NS
Mean number of risks	0.53	1.3	*P *< 0.0001
Source of funding NOT specified	83.5%	81.6%	NS
Conflicts of interest NOT specified	96.6%	96.0%	NS
At least one scientific/technical error in reporting on the trial	99%	99%	NS
Duration of the clinical trial NOT specified	59.7%	57.2%	NS
Sample size NOT specified	41.5%	29.4%	*P *< 0.0001
Dose NOT specified	81.0%	95.5%	*P *< 0.0001
Location of trial NOT specified	32.1%	32.3%	NS
Randomization NOT specified	89.2%	94.0%	NS
Double-blinding NOT specified	90.3%	98.5%	*P *< 0.0001
Use of placebo NOT specified	45.7%	72.6%	*P *< 0.0001
Withdrawals/dropouts NOT specified	98.0%	97.5%	NS

NS = not significant

Finally, we used logistic regression (multinomial for tone of assessment of the trial) adjusting for covariates that showed a significant difference using Wilcoxon tests to compare the measures of tone between herbal remedies and pharmaceuticals for clinical trials and newspaper articles. Results are presented as odds ratios and 95% confidence intervals where appropriate. All analyses were done in Stata version 7.0 (Stata Corporation, College Station, US).

### Methodological limitations

Our methods have a number of limitations. First, we surveyed only the print media, while television and increasingly the Internet are likely important sources of information for the public. We did, however, survey a broad range of newspapers of varying quality. Second, our study was limited to the small subset of newspaper stories that were directly related to peer-reviewed clinical trials. However, this met our objective to assess whether media coverage of the evidence-based trend in CAM was qualitatively different from reporting on conventional medicine with a long-standing tradition of clinical trial research. Finally, our study is a content analysis using an *a priori *coding frame [18-21], not a more detailed qualitative textual analysis. This enabled us to survey a larger number of clinical trials and newspaper articles and to test for statistically significant differences in reporting trends.

## Results

We coded 105 clinical trials (48 pharmaceutical, 57 herbal remedy) and 553 associated newspaper articles (201 pharmaceutical, 352 herbal remedy). The main comparisons are for tone (positive, neutral, negative), quality measures, and content between the herbal remedy and pharmaceutical clinical trials. There are four measures of tone: the tone of the results/outcomes of the clinical trial; the overall tone of the clinical trial, taking into account limitations and the discussion; the tone of the assessment of the specific clinical trial in the newspaper article; and the overall tone of the entire newspaper article.

### Clinical trials: herbal versus pharmaceutical

The clinical trials were published in 39 journals, primarily *JAMA*, the journal of the American Medical Association (21), the *Lancet *(14), *New England Journal of Medicine *(13), and *BMJ *(9). *JAMA *(10) and *BMJ *(8) published the greatest number of herbal remedy clinical trials reported in newspapers. Only one herbal remedy clinical trial was published in a CAM-specific journal, the *Journal of Alternative and Complementary Medicine*, confirming the trend for newspapers to report research in mainstream, high-impact medical journals. The majority of trials took place in North America, the UK and Europe, and the majority was conducted in public sector research institutions with only two pharmaceutical and four herbal remedy clinical trials taking place in the private sector.

**Figure 1:**
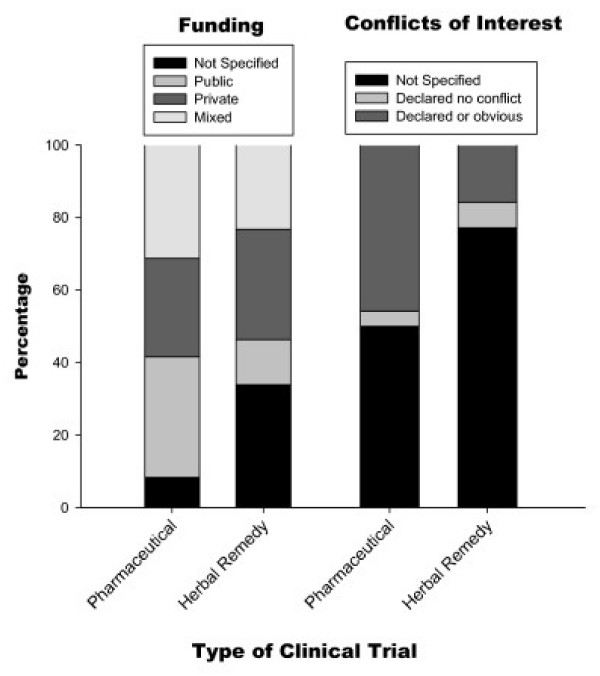
**Funding and conflicts of interest**. Comparison of the funding and conflicts of interest between herbal remedy clinical trials and pharmaceutical clinical trials.

The majority of pharmaceutical clinical trials were funded by the private sector (33%) or by mixed private and public funding (31%), but a minority of trials reported authors with conflicts of interest (Figure 1). Conflicts of interest were either not specified (50%) or there was a declaration of no conflict (4%). Of the pharmaceutical clinical trials, 46% declared conflicts of interest by authors, or these were obvious from the publication, generally because one or more of the authors were employed by the company that funded the trial (Figure 1). In comparison, 34% of herbal remedy clinical trials did not specify the source of funding. Those that did were funded by the private sector (30%) or through mixed public and private funding (23%). Very few herbal remedy clinical trials specified any further conflicts of interest; conflicts were either declared or obvious in 23%.

**Figure 2:**
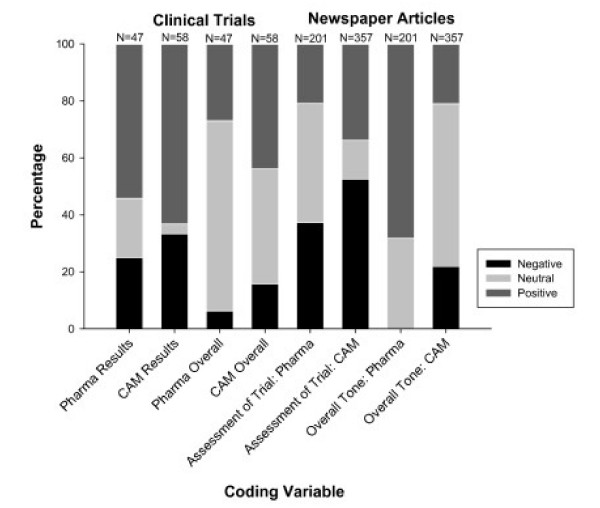
**Tone of clinical trials and related newspaper articles**. Comparison for the tone (positive, neutral, negative) of herbal remedy and pharmaceutical clinical trials and the newspaper articles reporting on those trials. There are four measures of tone: the tone of the results/outcomes of the clinical trial; the overall tone of the clinical trial, taking into account limitations and the discussion; the tone of the assessment of the specific clinical trial in the newspaper article; and the overall tone of the entire newspaper article.

There was a significant association between tone of clinical trial outcomes and type of clinical trial (*P *= 0.0201; *χ*^2 ^= 7.8129; degrees of freedom (*DF*) = 2) and between overall tone and type of clinical trial (*P *= 0.0230; *χ*^2 ^= 7.5462; *DF *= 2) (Figure 2). Most trials had a positive valuation of outcome, meaning that the trials were reporting positive results. However, a greater proportion of CAM clinical trials reported negative results (Figure 2). These associations are no longer significant using multinomial logistic regression after adjusting for significant covariates: length of trial and sample size (Table 1).

### Newspaper articles: herbal versus pharmaceutical

We coded articles from 131 newspapers in five countries. Newspapers with more than ten articles on herbal remedy clinical trials were the *Daily Mail *(UK), the *Kitchener-Waterloo Record *(Canada), the *New York Times *(US), the *Washington Post *(US), *Pittsburgh Post-Gazette *(US), and *The Record *(US). The majority of newspaper articles were published in the US (296), followed by the UK (151), Canada (100), and Australia/New Zealand (6). Most newspaper articles were reporting on the latest news (60%) and one third had a longer, investigative reporting format where the clinical trial was only one part of the article. The predominance of straight news reporting was reflected in whether the article was framed as a controversy: 43% of articles had no controversial element, 35% were framed as a balanced controversy, and 22% as an imbalanced controversy.

The main theme of almost all the articles on pharmaceutical clinical trials was the trial itself. This contrasted with articles on herbal remedy clinical trials where 63.6% focused on the trial and the other third focused on the medical condition, the myriad of uses for an herb, and the health risks associated with herbal remedies. The main benefit cited in almost all articles (90%) was improved health or treatment options, and only 4% cited no benefits. However, 54% of articles did not quantify the likelihood of the benefit. Interestingly, 29% of articles on herbal remedy versus 4% of pharmaceutical clinical trials stated there was no benefit. The main individual interviewed or quoted in the articles for both pharmaceutical clinical trials (63%) and herbal remedy clinical (73%) trials was a university or hospital scientist or physician not specializing in CAM. Indeed CAM researchers or practitioners were cited in only 8% of articles on herbal remedy clinical trials.

There were strong associations in newspaper articles between the tone of valuation of the results of the clinical trial and the type of trial (*P *< 0.0001; *χ*^2 ^= 135.5; *DF *= 2) and the overall tone of the newspaper article and the type of trial (*P *< 0.0001; *χ*^2 ^= 56.1; *DF *= 2). Newspaper articles were more highly polarized with respect to evaluating the outcomes of herbal remedy clinical trials than for pharmaceutical clinical trials. For pharmaceutical trials, the evaluation was most likely to be positive or neutral (Figure 2), while a greater proportion of newspaper articles evaluated the outcomes of herbal remedy clinical trials negatively.

The difference in overall tone of the newspaper article, where the clinical trial may have been only one part of a larger story was even more pronounced. The overall tone of the newspaper article was most likely to be positive (68.2%) or neutral (31.8%) when reporting on pharmaceuticals. Indeed no articles on pharmaceutical clinical trials had a negative overall tone. In contrast, 21.9% of articles on herbal remedy clinical trials had an overall negative tone, while the majority were neutral (57.1%), and 21% were positive (Figure 2). This is so, even though the articles reported significantly more risks, primarily adverse effects, associated with pharmaceuticals than herbal remedies (Figure 2). However, 40% of articles overall did not specify a risk, and 53% of those which did specify a risk did not quantify the likelihood of that risk. There were no significant associations between type of trial and conflicts of interest and type of funding of the trial; 83% of articles did not mention the source of funding for the trial and 96% did not specify the involvement of the funder. Similarly, 96% did not report on conflicts of interest. However, of the 29 articles that did report on conflicts of interest, 38% viewed the conflict negatively while 48% were neutral in their evaluation of the conflict.

The significant associations for overall tone remained when we used logistic regression and adjusted for significant covariates, year, and total number of risks. We calculated significance of overall tone for positive and neutral valuation categories only because one cell (negative valuation of pharmaceutical clinical trials) had zero counts (odds ratio = 13.210; 95% Wald confidence limits 7.414–23.539; *P *< 0.0001). We present the probabilities for tone of assessment of clinical trial outcomes adjusted for significant covariates because of the difficulties in interpreting multinomial logistic regression outputs (type of clinical trial: *P *< 0.0001; *χ*^2 ^= 41.26; *DF *= 2; year: *P *< 0.0001; *χ*^2 ^= 85.84; *DF *= 182; total risks: *P *< 0.0001; *χ*^2 ^= 19.19; *DF *= 2).

For all newspaper articles, there is a significant association between the type of medical condition and both of the measures of tone (overall: *P *< 0.0001, *χ*^2 ^= 9.5, *DF *= 12; tone of assessment of the trial: *P *< 0.0001, *χ*^2 ^= 41.7, *DF *= 12). The largest categories of medical conditions were diseases of the genitourinary system (mainly erectile dysfunction and menopause), a range of diseases affecting organ systems and mental disorders (mainly depression). Proportionally, the most negative overall tone was reserved for disorders associated with diet and obesity. The tone of assessment of the trial itself was proportionally most negative for diseases of the genitourinary system (mainly erectile dysfunction and menopause), mental disorders (mainly depression), and infectious diseases (mainly HIV/AIDS).

## Discussion

As CAM use increases alongside concerns about evidence of efficacy, we are interested in how the media, a significant source of medical information for the lay public and some professionals, report on clinical trials. This is the first study to compare systematically newspaper coverage of clinical trials for herbal remedies, a popular type of CAM, with clinical trials for pharmaceuticals. There has been a large increase in the number of clinical trials for herbal remedies over the last 20 years [23], the vast majority published in specialized journals on traditional Chinese medicines [24]. There has not, however, been a concomitant increase in the number of media articles reporting on clinical trials for herbal remedies; overall the media is not reporting on the trend toward evidence-based herbal medicine [23].

Those herbal remedy clinical trials that receive newspaper coverage are of similar quality to pharmaceutical clinical trials, likely reflecting the media's preference towards studies conducted in Western countries [24], and a select number of high-ranking journals for most coverage of medical research [25]. Our Jadad scores are marginally higher than those calculated for a greater variety of CAM trials published in a broader range of journals [24]. Another study comparing herbal remedy and pharmaceutical clinical trials conducted in Western countries also found no difference in quality of described methods [25]. Assessing reported methodology within clinical trials, however, may be an incomplete proxy for quality since methodology is often significantly abbreviated or published in accompanying longer articles [25]. In that case, sample size and duration of the study could be a more precise measure of trial quality [25], in which case, pharmaceutical clinical trials are significantly larger; many herbal remedy clinical trials had fewer than 100 patients and were of short duration.

Our results suggest high-impact journals publish a significant number of CAM studies with negative results [26,27], even though the general trend toward favoring positive results applies to both types of clinical trial [28]. Overall, however, the tone of both the positive and negative clinical trials is tempered in the discussion by highlighting, for example, trial limitations or conversely, potential for future research.

Despite the overall positive results and tone of the clinical trials, newspaper coverage of herbal remedy clinical trials was more negative than for pharmaceutical clinical trials. This is contrary to most published research on media coverage of CAM. Those studies consider a much broader spectrum of treatments and the media content is generally anecdotal rather than evidence based [29]. Indeed, journalists are displaying a degree of skepticism rare for medical reporting [23]. It is possible that once confronted with actual evidence, journalists are more critical or skeptical. It may be considered more newsworthy to debunk commonly held beliefs and practices related to CAM, to go against the trend of positive reporting in light of evidence. It is also possible that journalists who turn to press releases of peer-reviewed, high-impact journals have subtle biases towards scientific method and conventional medicine. Also, journalists turn to trusted sources in the biomedical community for comments on clinical trials, both herbal and pharmaceutical, potentially leading to a biomedical bias in reporting trial outcomes. Finally, the minority of newspaper articles were written by named journalists. Since it was not possible to identify the authors of the majority of newspaper stories, it is possible that some journalists with either a positive or negative bias contributed disproportionately to the news coverage.

### Implications of results

Despite repeated studies, the media continues to provide insufficient information to the public largely through omission, an under-reporting of risk, and a lack of disclosure of trial funding and potential conflicts of interest. The latter is true for both pharmaceuticals and herbal remedies but may be more critical for herbals, which may be accessed by the lay public without a physician intermediary.

Media coverage is not providing the public with the information necessary to make informed decisions about medical treatments, either conventional or alternative. There are significant errors of omission of basic information such as dose, sample size, location and duration of the trial, and methods for randomized clinical trials. In addition, there is an under-reporting of risks, especially in the context of herbal remedies. This is common in the context of medical reporting where difficulties stem from a lack of comprehension, both by the public and journalists, of differences between absolute and relative risks and the nature of probabilistic analyses [30]. Many studies have reported on such errors of omission [18-20], citing space and time constraints of medical journalists whose stories compete for publication and the attention of editors with general news and other content [19,31].

The media is also overly reliant on narratives from satisfied patients, researchers, clinicians, and patient groups without disclosing financial ties to industry and conflicts of interest [20,21,32]. 'Conflicts occur when scientists are expected to exercise judgment dispassionately but instead are motivated by financial, professional, or other types of interest' [33]. There has been an international movement toward reporting of funding and conflicts of interest in published clinical trials, yet a significant portion of herbal remedy clinical trials in our study did not disclose any funding source. Those that did showed substantial funding from the private sector, not surprising considering the current economics of CAM and interest in CAM by pharmaceutical companies. Even more concerning was the lack of disclosure of conflicts of interest, either positive or negative, for both types of clinical trials. This suggests the need for better implementation of funding and conflicts of interest disclosure policies by medical journals [34].

The media should be encouraged to disclose the interests of researchers and institutions involved in clinical trials that could influence the outcome or reporting of trial results [33]. Without such information, which our study shows is largely missing from media coverage, it is not possible for the lay public to assess the credibility of the research. This lack of reporting should be of great concern to the medical research community, which has the most to lose by way of public trust and respect. Academic researchers and institutions have the most to gain from the development and enforcement of full disclosure guidelines for both medical publications and for the media.

Overall concerns have lead to suggestions for minimum standards and content for medical coverage [32]. Gary Schwitzer and colleagues [32] have argued ' [j]ournalists have a special responsibility in covering health and medical news' knowing that 'readers and viewers may make important health-care decisions based on the information provided [in media stories]'. In response, solutions must be aimed at all three major stakeholders and involve guidelines for journalists [35] and increased training in health and science journalism [20], increased training in media communication skills for clinical researchers [36-38], and scientific and media literacy programs for the lay public [39]. Given the well-established and expanding market for health coverage, it is time for journalists and editors to experiment with improving content without necessarily sacrificing narrative themes such as human-interest stories. A change for the better is unlikely to result in a reduced public appetite for health news – an appetite that is increasingly sophisticated and desirous of high quality information [40].

## Abbreviations

CAM: complementary and alternative medicine; DF: degrees of freedom.

## Authors' contributions

All authors contributed to the conception and design of the study. TB co-ordinated the data collection and coding, TB and HB in consultation with Rahim Moineddin performed the statistical analyses, and TB wrote the first draft. All authors were involved in the writing of the final draft. TB is guarantor for the study.

## Appendix

### Appendix 1: Coding frame for clinical trials

1. Basic information

   a. Trial number

   b. Type of clinical trial   (Pharmaceutical = 1; CAM = 2)

   c. Journal name

   d. Year

   e. Type of institution where research conducted (lead or corresponding author)

         Not specified               0

         University/hospital            1

         Not-for-profit organization         3

         Mixed                  4

         Private                  5

         Government               6

2. Contents

   a. Medical condition

         Not specified                     0

         Type of medical condition

   b. Dose(s) specified (0 = no; 1 = yes)

   c. Location of clinical trial

            Not specified      0

            US         1

            Canada         7

            UK         2

            Europe         3

            Australia      4

            South America   5

            Asia         6

            International      8

            Mid East/Africa   9

   d. Is the article framed as a controversy? (no)         1

      If yes, is the report

         balanced             2

         or imbalanced            3

   e. Type of main benefit

      Not specified                  0

      None (stated that there is no benefit)         1

      Basic research                  2

      Improved health/treatment            3

      Decreased side-effects            4

      General safety                  5

      Increased autonomy/empowerment         6

      Spiritual, moral, ethical            7

      Environmental/ecological/nature         8

      Economic                  9

      Improved quality of life            10

      Other (specify)               11

   f. Likelihood of benefit

      Not specified                  0

      High                     1

      Moderate                  2

      Low                     3

      No benefit (stated)               4

      Mentioned but not quantified            5

   g. Total number of benefits mentioned            number

   h. Type of main risk/cost

      Not specified                  0

      None (stated that there is no risk)         1

      Basic research                  2

      Health                     3

      Increased side-effects               4

      General safety                  5

      Decreased autonomy/empowerment         6

      Spiritual, moral, ethical            7

      Environmental/ecological/nature         8

      Economic                  9

      Decreased quality of life            10

      Other (specify)               11

   i. Likelihood of risk/cost

      Not specified                  0

      High                     1

      Moderate                  2

      Low                     3

      No risk/cost (stated)               4

      Mentioned but not quantified            5

   j. Total number of risks/costs mentioned         number

3. Quality of clinical trial

   a. Was the study described as randomized (this includes the use of words such as randomly, random and randomization? (no = 0; yes = 1)

   b. Was the study described as double blind? (no = 0; yes = 1)

   c. Was there a description of withdrawals and dropouts? (no = 0; yes = 1)

   d. What was the Jadad score for quality of clinical trial? (score 1–5)

   e. What was the sample size?

   f. What was the length of the clinical trial (days)?

4. Conflicts of interest

   Not specified                     0

   Mentioned but none                  1

   Declared or obvious                  2

5. Funding of the trial

   Not specified                     0

   Public                        1

   Private                        2

   Mixed                        3

6. Judgments and ratings

   a. Tone of clinical trial outcome

      Negative valuation of results         1

      Positive valuation of results         2

      Neutral valuation of results         3

   b. Overall tone of discussion/conclusions, taking into account study limitations

      Negative valuation            1

      Positive valuation            2

      Neutral valuation            3

Explanation of coding frame for newspaper articles

7. Basic information

   a. Trial and newspaper number

   b. The name of the newspaper/country of newspaper

      Australia/New Zealand   1

      Canada            2

      UK/Ireland         3

      USA            4

   c. Year of the newspaper article

8. Attention structure

      With these variables we are measuring the editorial importance of an article; the means used to attract the reader's attention.

      a. Word count

      b. News format

      (here we are attempting to distinguish between facts and opinion)

         Not specified                        0

         Article with latest news                  1

         Investigation, reportage, background               2

         Interview (mainly)                     3

         Column, commentary by regular columnist            4

         Editorial (paper's editor)                  5

         Commentary from other people (e.g. politicians, religious leaders, special interest groups)                  6

         Letters to the editor                     7

         Review of books, films, etc.                  8

         Other                           9

9. Contents

   a. Main theme

   Science/medicine

      Clinical trial                     1

      Meta-analysis of clinical trials               2

      Medical condition                   3

      Review of treatments for a medical condition         4

      Review of uses of a particular herb            5

   Safety/risks

         Health risks: CAM                   6

         Health risks: traditional medicine                7

         Other risks: CAM                     8   

         Other risks: traditional medicine                9

   Other issues

         Patenting, property rights                   10

         Economic prospects, opportunities             11

         Biopharmaceutical industry                12

         CAM industry                      13

         Biodiversity/conservation                   14

         Legal/regulatory                      15

         Science policy                      16

         Education                         17

         Public opinion (e.g. survey results)             18

         Public protest/demonstration                19

         Ethical issues                      20

         Religious issues                      21

   b. Name of herbal remedy/remedies or name of pharmaceutical/traditional medicine(s)

      (no = 0; yes = 1)   

   c. Dose(s) tested (no = 0; yes = 1)   

   d. Use specified (no = 0; yes = 1)

   e. Main voice (who/what is the main spokesperson/group/institution quoted or described)

      Not applicable, unknown                      0

      Public sector

         Parliament/congress                      1

         Government

            General                         2

            Health                         3

            Industry                         4

            Environment                         5

            Patent Offices                      6

         Government research institutions/scientists (e.g. National Institutes of Health)                      7

         Specialist CAM researcher/practitioner                8

         Non-CAM university or hospital scientists/physicians         9

         Ethics committees                      10

         Judicial, legal voice                      11

         The public, public opinion (e.g. surveys)             12

         The media, published opinion                   13

         Celebrity (sports, film TV)                   14

      Private sector – business

         Scientists in private laboratories                15

         Pharmaceutical company/spokesperson                16

         CAM company spokesperson                   17

         Venture capital                         18

         Private investors                        19

         Stock Exchange                         20

      Private sector – other

         Political parties                         21

         Religious organizations                      22

         Consumer groups                      23

         Patient groups/lobbies                      24

         Environmental organizations                   25

         Professional organizations (medical, legal, etc.)          26

      International institutions

         Developing countries                      27

         European Union                         28

         European Parliament                      29

         United Nations organizations                   30

         Other international organizations                31

   f. Location of clinical trial stated (no = 0; yes = 1)

   g. Is the article framed as a controversy? (no)            1

      If yes, is the report

         balanced             2

         or imbalanced            3

   h. Type of main benefit

      Not specified                  0

      None (stated that there is no benefit)         1

      Basic research                  2

      Improved health/symptoms/condition      3

      Decreased side-effects            4

      General safety                  5

      Increased autonomy/empowerment         6

      Spiritual, moral, ethical            7

      Environmental/ecological/nature         8

      Economic                  9

      Improved quality of life             10

      Other (specify)               11

   i. Likelihood of benefit

      Not specified                  0

      High                     1

      Moderate                  2

      Low                     3

      No benefit (stated)               4

      Mentioned but not quantified            5

   j. Total number of benefits mentioned               number

   k. Type of main risk/cost

      Not specified               0

         None (stated that there is no risk)         1

         Basic research                  2

         Health/symptoms/condition            3

         Increased side-effects               4

         General safety                  5

         Decreased autonomy/empowerment         6

         Spiritual, moral, ethical            7

         Environmental/ecological/nature         8

         Economic                  9

         Decreased quality of life            10

         Other                     11

   l. Likelihood of risk/cost

      Not specified                  0

      High                     1

      Moderate                  2

      Low                     3

      No risk/cost (stated)               4

      Mentioned but not quantified            5

   m. Total number of risks/costs mentioned               number

10. Conflicts of interest

   Not specified                     0

   Mentioned but none                  1

   Declared or obvious                  2

11. Funding of the trial

   Not specified                     0

   Public                        1

   Private                        2

   Mixed                        3

12. Involvement of funding agency

   Not specified                     0

   Specified                     1

13. How conflict of interest is viewed

   Not mentioned                     0

   Negative                     1

   Positive                     2

   Neutral                        3

14. Judgments and ratings

   a. Tone of assessment of clinical trial outcomes

         Negative valuation of results         1

         Positive valuation of results         2

         Neutral valuation of results         3

   b. Frame (choose the one that best describes the article)

      1. Descriptive (a purely descriptive account of clinical trial with little or no context outside the technology/research)

      2. Descriptive with context (a descriptive account of clinical trial placed in context of other research, history, medical condition, herbal remedy, etc.)

      3. Progress (a celebration of new development; breakthrough; direction of history; conflict between progressive/conservative-reactionary)      

      4. Economic prospect (economic potential; prospects for investment and profits; R & D arguments)   

      5. Dissatisfaction   (patients are dissatisfied with conventional treatment because it has been ineffective, has produced adverse effects, or is seen as impersonal, too technologically oriented, and/or too costly)

      6. Need for personal control (patients seek alternative therapies because they see them as les authoritarian and more empowering and as offering them more personal autonomy and control over their health-care decisions)

      7. Philosophical congruence (alternative therapies are attractive because they are seen as more compatible with patients' values, worldview, spiritual/religious philosophy, or beliefs regarding the nature and meaning of health and illness)

      8. Ethical (call upon ethical principles; thresholds; boundaries; distinctions between acceptable/unacceptable risks in discussions on known risks; dilemmas; professional ethics

      9. Risks before the event (call for restraint in the face of unknown risk; warning; unknown risks as anticipated threats; catastrophe warnings)   

      10. Risks after the event (fatalism after the innovation; having adopted the new technology/products a price may well have to be paid in the future; no control any more after the event)   

      11. Public accountability (call for public control; participation; public involvement; regulatory mechanisms; private versus public interests; openness of procedures; transparency; justification of procedures)

      12. Globalization (call for global perspective; national competitiveness within an economy or isolationism)

      13. Profile/human interest story

15. Accuracy

This section examines the accuracy of the newspaper article in its reporting of the trial.

   a. What/who is cited as the main source of the information?

      The clinical trial journal article               1

      The authors of the clinical trial               2

      Other scientists/physicians/practitioners            3

      A press release/conference               4

      A company spokesperson                  5

      A secondary source (e.g. other newspaper/review article)   6

      Celebrities                        7

      Other commentators (please specify)            8

      Not specified                     0

   b. Was the study described as randomized (this includes the use of words such as randomly, random and randomization?

      Not specified      0

      Yes, accurately   1

      Yes, inaccurately   2

   c. Was the study described as double blind?

      Not specified      0

      Yes, accurately   1

      Yes, inaccurately   2

   d. Was use of placebo mentioned?

      Not specified      0

      Yes, accurately   1

      Yes, inaccurately   2

   e. Was there a description of withdrawals and dropouts?

      Not specified      0

      Yes, accurately   1

      Yes, inaccurately   2

   f. Was the sample size mentioned?

      Not specified      0

      Yes, accurately   1

      Yes, inaccurately   2

   g. What was the length of the clinical trial mentioned?

      Not specified      0

      Yes, accurately   1

      Yes, inaccurately   2

   h. Are there any significant technical/scientific errors in the reporting? (Assume that there are no errors in the scientific journal article)

      None                     1

      1–3                     2

      >3                     3

   i. Overall, do the main claims made in the newspaper article accurately reflect the research findings?

      Negative               1

      Neutral                  2

      Positive               3

## Pre-publication history

The pre-publication history for this paper can be accessed here:


